# Evaluation of a novel metric for personalized opioid prescribing after hospitalization

**DOI:** 10.1371/journal.pone.0244735

**Published:** 2020-12-31

**Authors:** Nicholas R. Iverson, Catherine Y. Lau, Yumiko Abe-Jones, Margaret C. Fang, Kirsten N. Kangelaris, Priya Prasad, Sachin J. Shah, Nader Najafi

**Affiliations:** Department of Medicine, University of California, San Francisco, San Francisco, California, United States of America; University of South Australia, AUSTRALIA

## Abstract

**Background:**

The duration of an opioid prescribed at hospital discharge does not intrinsically account for opioid needs during a hospitalization. This discrepancy may lead to patients receiving much larger supplies of opioids on discharge than they truly require.

**Objective:**

Assess a novel discharge opioid supply metric that adjusts for opioid use during hospitalization, compared to the conventional discharge prescription signature.

**Design, setting, & participants:**

Retrospective study using electronic health record data from June 2012 to November 2018 of adults who received opioids while hospitalized and after discharge from a single academic medical center.

**Measures & analysis:**

We ascertained inpatient opioids received and milligrams of opioids supplied after discharge, then determined days of opioids supplied after discharge by the conventional prescription signature opioid-days (“conventional days”) and novel hospital-adjusted opioid-days (“adjusted days”) metrics. We calculated descriptive statistics, within-subject difference between measurements, and fold difference between measures. We used multiple linear regression to determine patient-level predictors associated with high difference in days prescribed between measures.

**Results:**

The adjusted days metric demonstrates a 2.4 day median increase in prescription duration as compared to the conventional days metric (9.4 vs. 7.0 days; *P*<0.001). 95% of all adjusted days measurements fall within a 0.19 to 6.90-fold difference as compared to conventional days measurements, with a maximum absolute difference of 640 days. Receiving a liquid opioid prescription accounted for an increased prescription duration of 135.6% by the adjusted days metric (95% CI 39.1–299.0%; *P* = 0.001). Of patients who were not on opioids prior to admission and required opioids during hospitalization but not in the last 24 hours, 325 (8.6%) were discharged with an opioid prescription.

**Conclusions:**

The adjusted days metric, based on inpatient opioid use, demonstrates that patients are often prescribed a supply lasting longer than the prescription signature suggests, though with marked variability for some patients that suggests potential under-prescribing as well. Adjusted days is more patient-centered, reflecting the reality of how patients will take their prescription rather than providers’ intended prescription duration.

## Introduction

Prolonged opioid prescriptions and higher daily doses of opioids have been associated with significantly increased risk of development of chronic opioid therapy, opioid use disorder, and opioid overdose [1–3]. Prior studies have described wide variation in post-hospitalization prescribing of opioids [4–9]. However, prior work is limited because it does not account for patients’ actual opioid needs in the hospital when describing discharge opioid prescribing practices.

Opioid-days prescribed on discharge from the hospital has conventionally been characterized by the prescription signature—the total number of pills dispensed divided by the number of pills per day. Although this method may allow for ease of calculation of intended prescription duration, it does not account for what a patient actually required during their hospitalization and thus may not reflect how long a patient’s prescription will truly last once they leave the hospital. This could lead to patients receiving excessive opioid supplies after discharge.

In this study, we sought to assess whether adjusting duration of patients’ discharge opioid prescriptions based on individual opioid needs during hospitalization demonstrates a difference in opioid-days prescribed as compared to the conventional discharge prescription signature. We hypothesize that many patients are discharged with opioids despite requiring none in the 24 hours prior to discharge, and that many patients receive a much larger discharge supply than they may require after adjusting for their inpatient opioid needs.

## Methods

### Study design & patient selection

This retrospective observational study used electronic health record data to identify all patients discharged from an acute care inpatient medicine service at a single tertiary care academic medical center (University of California, San Francisco) from June 1, 2012 to November 30, 2018. We included all patients who had received opioids during their hospitalization and at discharge for the primary analysis since these metrics apply to all patients discharged from the hospital. For the purposes of this study, we excluded patients with ICD-9 or ICD-10 codes for sickle cell disease or cancer-related pain, who were on hospice before or after discharge, were provided comfort care measures, or who were followed by the inpatient palliative care service. We excluded these groups of patients despite the metrics theoretically still applying to these groups because the goals of their pain control are often different than all-comers to the internal medicine service. For patients with sickle cell disease, the medical center often has pre-determined pain medication plans in place and their pain medication doses are often much higher and controlled differently than for other patients. Similarly, for patients who have cancer-related pain, are on hospice or are being seen by palliative care, the use of a metric that seeks to identify over- or under-prescription of opioids seems less clinically applicable in this population where pain control goals may be highly variable, though mathematically possible. We also excluded discharge opioid prescriptions that were a resumption of the patient’s prior to admission opioid prescription, since it does not reflect a prescribing decision by the inpatient provider.

### Measures of opioid prescribing

**Fig 1:**
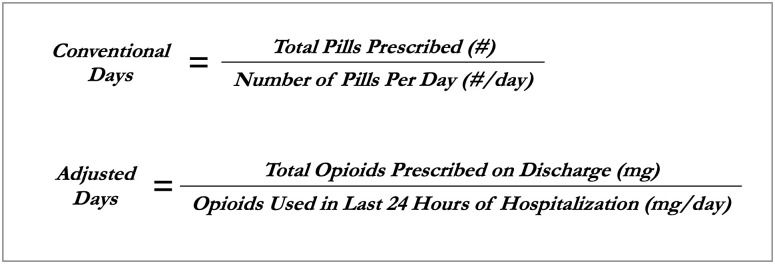
Formulas for calculating opioid prescription duration using the conventional metric and the novel hospital-adjusted metric.

All opioid prescriptions were standardized to morphine milligram equivalents (MMEs) based on published conversion ratios [10]. For each hospital day, we ascertained the total MME of opioids administered—including short and long acting opioids as well as all opioid formulations. The number of opioid-days supplied on discharge was first determined using the “conventional days” metric. This metric is calculated from the discharge prescription signature, defined as the total quantity of doses prescribed divided by the number of doses per day (Fig 1). For example, if a patient was discharged with morphine 15 mg tablets every 4 hours as needed (6 tablets per day), dispense #42, that patient received a 7 day supply. Next, the number of opioid-days supplied on discharge was determined for the novel “adjusted days” metric, defined as the number of total MME of opioids supplied at discharge divided by the total MME administered to the patient during the last 24 hours of hospitalization (i.e. mg/mg per day). For example, if the patient that received the discharge prescription above had only taken one 15 mg tab of morphine in the 24 hours prior to discharge, they would have received an opioid supply of 42 days by adjusted days (630 total MME supplied on discharge, divided by 15 MME actually required per day). We chose the last 24 hours to balance capturing a length of time as close to discharge as possible in order to most closely reflect how patients may actually take their opioids after discharge, since opioid usage can vary throughout a hospitalization and generally declines near discharge, while choosing a reasonably long enough time such that we would expect to capture patients’ need for and use of opioids if still requiring them while in the hospital. If a patient is discharged with prescriptions for multiple opioids, the longest single prescription duration was used. Delta days were defined as the difference in days prescribed between the metrics, and were calculated by subtracting conventional days from adjusted days.

### Statistical analysis

Statistical analyses were performed with Stata, version 15.0 (StataCorp). We performed descriptive statistics and used the sign test to compare days supplied between the two metrics, with statistical significance defined as an alpha of <0.05. To quantify the difference in opioid days determined by the two methods of measurement, we calculated the within-subject difference and within-subject mean for each patient [11]. When we initially plotted these values, we noted that the difference rose with the mean (i.e. the variability rose with the magnitude of the measurements), which is common in biomedicine and precludes summarizing the relationship of the two measurements with a single mean difference. A log transformation of the data eliminated this association of magnitude of within-subject difference and within-subject mean, allowing for calculation of mean and standard deviation of within-subject difference that does not vary with magnitude [12] (S1 Fig). Due to the log transformation, the overall comparison of opioid days methods must now be reported as a ratio (fold change from conventional days to adjusted days) in which 95% of all within-subject pairs of measurements are expected to fall.

We used multiple linear regression to determine significant predictors associated with delta days. We similarly log transformed delta days to ensure homoscedasticity and normal distribution of residuals, and thus regression results are reported as a percent change in delta days. We performed analysis to ensure assumptions of multiple linear regression were met (linearity, co-linearity, normal distribution of residuals, homoscedasticity). Log transformation reduced the heteroscedasticity caused by outliers and allowed our model to meet the regression assumptions. Under those conditions, we did not omit any measurements from our analyses because extreme values were still important to the conclusions of our study, as the patients with the highest opioid days by either metric add value to our understanding of the difference between the metrics.

Our model included demographic data on patient age; gender (limited to male and female because of retrospective EMR data); limited English proficiency, defined as need for interpreter use; and race/ethnicity, with white used as the reference category. Mental health diagnoses were obtained by ICD-10 code, including mood disorders (F30-34), anxiety disorders (F41), post-traumatic stress disorder (F43.1), and non-mood psychotic disorders (F20-29). Hospitalization characteristics included any stay in the intensive care unit (ICU); discharge location, categorized as home or self-care (used as the reference category), home with home health care, monitored non-hospital facility (including skilled nursing facilities, acute rehabilitation facilities, assisted living/intermediate care facilities, inpatient psychiatric facilities, and long term acute care facilities), other acute care hospital, and discharge against medical advice; discharge service type, categorized into teaching services or hospitalist-only services; and average daily MME, to determine if higher inpatient opioid requirement are associated with differences in the metrics on discharge prescribing. We also included days until follow up appointment, AHRQ mortality and re-admission indices, benzodiazepine use on admission, prescription of a liquid opioid on discharge, and time. The time variable, as a sequence of month-years, was included due to increased national attention on the opioid crisis to control for and determine if there was a significant change in delta days over time and evaluate current relevance of the adjusted days metric.

In a secondary analysis, we sought to analyze a subset of patients identified by our metric who likely inappropriately received a new opioid prescription on discharge: patients who were not on opioids prior to their hospitalization who received opioids while hospitalized but not in the 24 hours prior to discharge, yet still received a prescription for new opioids on discharge. We used logistic regression to determine predictors associated with receipt of an opioid prescription on discharge, and thus also included the subset of patients with these same characteristics but who did not receive an opioid prescription on discharge in our study population for this secondary analysis. The same covariates in the model above were used, with the exclusion of liquid opioid prescription and days until follow up appointment, since neither apply to the subset of patients that did not receive an opioid prescription.

### IRB approval

The UCSF Institutional Review Board for Human Subjects Research approved this study.

## Results

### Patient characteristics

Of 5,544 patients who met the inclusion criteria for the primary analysis, 2,052 (37.0%) were not on opioids prior to admission (Table 1). An additional 325 patients were identified by our adjusted days metric who were not on opioids prior to admission and received opioids while hospitalized, but were discharged on opioids despite requiring no opioids in the 24 hours prior to discharge. This subset of our study patients was not included in the primary analysis since their adjusted days were incalculable (infinite, since the denominator of MME in the last 24 hours of hospitalization equals zero), but characteristics of this subgroup are analyzed independently in a secondary analysis, as compared to the 3,796 patients we identified during the same study period with the same characteristics but who did not receive an opioid prescription on discharge.

**Table 1:** Patient characteristics.

	Primary analysis population (n = 5,544)
Age, mean (SD)	53.0 (17.8)
Women	3,045 (54.9%)
Limited English proficiency	547 (9.9%)
Race/Ethnicity	—
• *White*	2855 (54.7%)
• *Black/African American*	946 (18.1%)
• *Latinx/Hispanic*	701 (13.4%)
• *Asian*	623 (11.9%)
• *American Indian or Alaska Native*	41 (0.8%)
• *Native Hawaiian or Other Pacific Islander*	56 (1.1%)
Benzodiazepines prior to admission	1,408 (25.4%)
Length of stay, mean (SD)	7.3 (9.8)
ICU stay during hospitalization	717 (12.9%)
Discharge from teaching service	4,221 (76.1%)
Discharge disposition	—
• *Home/self care*	3,046 (55.4%)
• *Home health care*	1,292 (23.5%)
• *Monitored non-hospital facility*	1,029 (18.7%)
• *Other acute care hospital*	100 (1.8%)
• *Against medical advice*	27 (0.5%)

Number and (%) of patients by category, unless otherwise noted.

### Prescribed opioid characteristics

Patients received a median of 70.7 MME per day during the hospitalization (IQR 31.1–168.9), and a median of 63.0 MME in the last 24 hours of their hospitalization (IQR 30.0–162.0). A median of 450.0 MME was supplied on discharge (IQR 200.0–1505.0).

**Fig 2:**
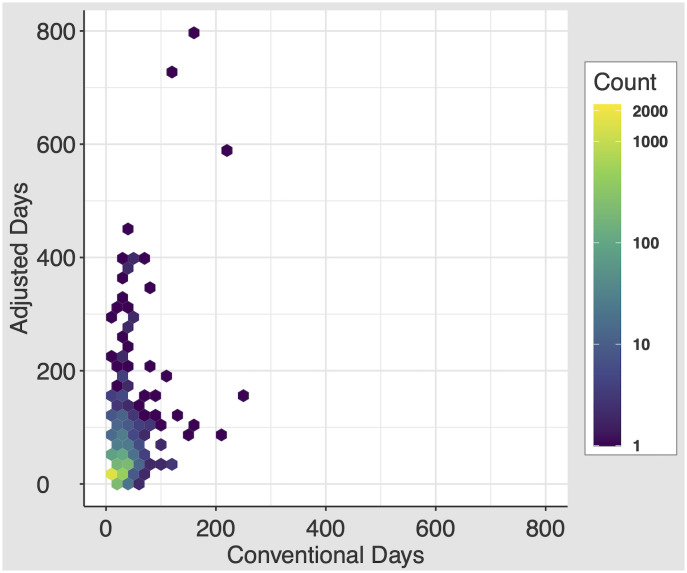
Distribution of the conventional days and adjusted days metrics. 2D histogram, with each equally-sized hexbin’s color demonstrating the concentration of patients in that bin.

On discharge, patients received a median conventional days of 7.0 days (IQR 4.0–15.0) and a median adjusted days of 9.4 days (IQR 3.8–20.0; *P*<0.001). The minimum and maximum number of conventional days prescribed are 0.0001 and 240 (Fig 2). The minimum and maximum number of adjusted days prescribed are 0.00001 and 800. By the adjusted days metric, 283 (5.1%) patients were prescribed less than one day of opioids on discharge. The minimum and maximum number of delta days are -126.2 and 640.

Patients who were on opioids at the time of admission were prescribed a median of 9.0 conventional days (IQR 5.0–20.0) and 10.0 adjusted days (IQR 4.4–22.5; *P* = 0.433). Patients not on opioids at the time of admission were prescribed a median of 5.0 conventional days (IQR 3.3–10.0) and 7.1 adjusted days (IQR 3.3–15.8; *P*<0.001).

### Method agreement between conventional and adjusted days measures

Mean and standard deviation of differences for all pairs of natural log-transformed opioid-days measures were 0.125 and 0.922, respectively (S1 Fig). After exponentiation (i.e. anti-log), the mean difference demonstrates that adjusted days are, on average, 1.13-fold higher than conventional days. Additionally, 95% of all adjusted days measurements are found within a 0.19- to 6.90-fold difference of the prescription duration by the conventional days metric.

### Patient-level predictors associated with large differences between the measures

**Fig 3:**
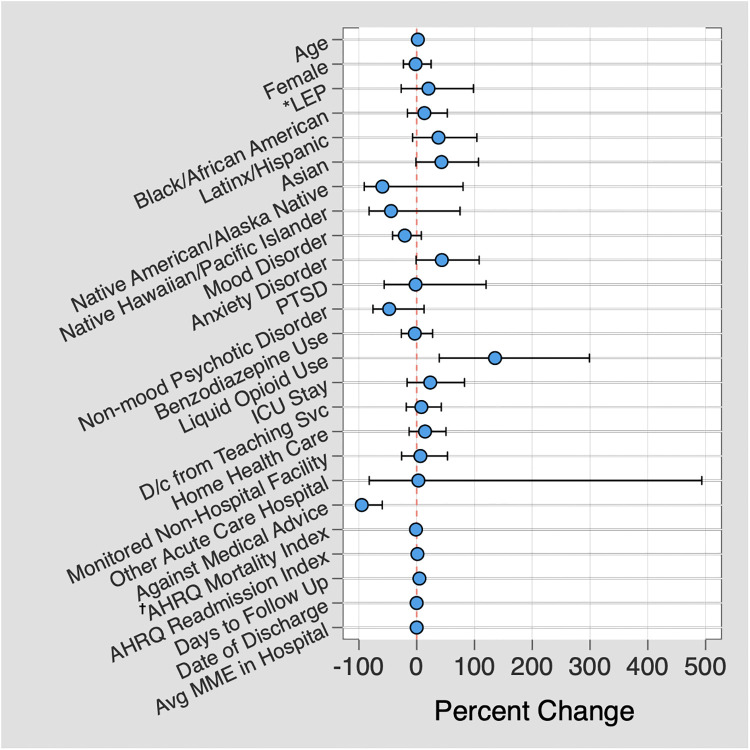
Predictors associated with high delta days. Multiple linear regression demonstrating predictors associated with a large difference in number of days prescribed between adjusted days and conventional days metrics, plotted against the percent difference between the two metrics. *LEP = Limited English proficiency; † AHRQ = Agency for Healthcare Research and Quality.

Regression analysis for patient factors significantly associated with increased delta days demonstrated two predictors with remarkably larger magnitudes of difference than other predictors: receipt of a liquid opioid prescription, with a 135.6% increase in delta days (95% CI 39.1–299.0%; *P* = 0.001), and leaving against medical advice, with a 95.1% decrease in delta days (95% CI -99.4– -59.6; Fig 3; S1 Table).

### Predictors of patients receiving a discharge opioid prescription despite not using them in the 24 hours or prior to discharge in patients without opioids on admission

These patients received a median of 9.9 MME per day while hospitalized (IQR 4.5–23.6). On discharge, they received a median of 160 MME (IQR 112.5–300.0) and a median of 5.0 days by conventional days (IQR 3.3–7.5). Adjusted days was infinite by our formula since MME in the last 24 hours was zero, allowing for identification of this population by the novel metric. Logistic regression evaluating predictors associated with receiving a discharge opioid prescription revealed significant associations with mood disorder diagnosis (OR 0.52 [0.32–0.83]; *P* = 0.007), non-mood psychotic disorder diagnosis (OR 0.28 [0.10–0.82]; *P* = 0.019), admission to the ICU (OR 0.43 [0.30–0.60]; *P*<0.001), discharge from a teaching service (OR 0.37 [0.29–0.48]; *P*<0.001), and discharge to either home with home health care (OR 1.63 [1.16–2.28]; *P* = 0.005) or to a monitored non-hospital facility (OR 3.15 [2.25–4.39]; *P*<0.001; S2 Table).

## Discussion

In this study of hospitalized internal medicine patients that evaluates differences in discharge opioid prescription duration after adjustment for actual opioid requirements while hospitalized, we found that the novel adjusted days measure demonstrated significantly more median days prescribed as compared to conventional days, with 95% of within-subject paired measurements expected to fall between a 0.19- and 6.90-fold difference between the measures. For a 7 day prescription by conventional days, this would indicate that 95% of adjusted days fall between 1.33 and 48.3 day prescription durations—a striking contrast to the conventional days metric. This likely reflects that physicians quickly calculating opioid needs in their head tend to prescribe more or less opioids than they intend to, but that the amount by which they over- or under-prescribe is not consistent in a way that allows us to, for example, just add a coefficient to conventional days in order to approximate adjusted days. The solution is to truly personalize the prescription to the patient.

While on average prescriptions were longer by the adjusted days measure, we can see from our 2D histogram that patients were prescribed extremely long durations of opioids by both measures, and that patients who are prescribed long durations by one measure are not always the same as the long durations by the other. This difference between provider intended prescription duration (conventional days) and expected duration of actual use after discharge (adjusted days) suggests that how a patient uses a prescription after discharge may not match the provider’s intended prescription. There were instances of delta days at both extremes—highly positive (longer lasting prescription than the provider prescribed, with the largest difference in days prescribed between the measures of +640 days) and negative (shorter prescription duration than the provider prescribed). Concerningly, this trend occurs especially in patients not on opioids at the time of admission. Given the importance of capturing prolonged opioid prescriptions, these results demonstrate ongoing room to improve our prescribing patterns for patients supplied long durations of opioid prescriptions on discharge from the hospital.

While it is important to identify patients at the highest risk for complications from opioid use and target prescribing for the shortest duration needed, it is important to recognize that this measure also has the potential to identify patients who may receive shorter prescriptions than providers intended based on their inpatient requirements as well. We found that some patients had large negative delta days, indicating a much shorter prescription than the patient’s inpatient requirements, and that 5.5% of patients in our study were prescribed a less than one day supply by adjusted days. Because the duration of this prescription is adjusted for the requirements during the last 24 hours of hospitalization, conditions allowing for a rapid taper of opioids after discharge will demonstrate a shorter prescription duration when using adjusted days. Thus, we are unable to capture true “under-prescribing” with this measure. However, a less than one day prescription based on the patients’ inpatient needs is likely less than providers intended and may represent true under-prescribing. While using this measure as a metric for under-prescription may be less reliable, it still can act as a starting point for patients for whom we may be under-prescribing needed pain control measures.

We found a strong association with high delta days and liquid opioid prescribing. We hypothesize this is due to the challenge of mentally converting milliliters of liquid opioid per dropper into the familiar milligrams per tablet to which clinicians are accustomed. However, it would require further research to understand if this is the true root cause. If this is the root cause, as we expect it is without having other likely explanations, this issue could be remedied easily with a built-in EMR opioid calculator and decision support tool. With regard to leaving against medical advice, the substantial decrease in delta days seen may be associated with patients who self-discharge receiving much shorter prescriptions (if any) than other patients, and thus may have less room for error or increased provider attention on prescription duration. The large difference between the two measures was not significantly associated with date of discharge in our regression, indicating that providers have not increasingly matched discharge opioid prescribing to inpatient need over time, despite the increased public and provider attention on opioid prescribing during this study period. This key negative result indicates the importance of implementing the novel adjusted days metric in routine practice, even now. Our study does not explore the causal pathways to explain the predictors in this multivariate analysis, but reasonable clinical explanations can be generated for the two strongest significant predictors as above. Furthermore, it is essential to acknowledge that the regression model does not account for all possible patient, provider, and environmental factors that are challenging to measure in a real-world clinical environment, and that these significantly associated predictors do not explain the complete picture of the differences seen between these measures. We hypothesize that much of the difference between the measures is due to unmeasured physician behaviors regarding EMR data review during discharge planning, by inadequately reviewing the patient’s opioid requirements when calculating discharge opioids to dispense.

As previously described, prolonged opioid-days prescribed has been associated with poor patient outcomes, with one of the sharpest increases in the risk of developing chronic opioid use after taking just 5 days of therapy, and again at 31 days [13]. Moreover, recent literature shows that more than 40% of adults who misuse opioids received them from family and friends [14]. Having excess opioids in the home contributes to both poor patient outcomes as well as the larger societal issue of opioid misuse, which is why we must recognize when discharge prescriptions may be in patients’ homes for longer. In addition, an unexpected result of measuring opioid days by adjustment for hospital need was that incalculable values flagged internal medicine patients who received a discharge prescription for opioids despite requiring no opioids in the 24 hours prior to discharge—a population previously undescribed in the literature. This prescribing practice puts patients at unnecessary risk for complications from opioid use. This could be both a high impact and low resource target for future hospital quality improvement initiatives.

This study has a number of notable strengths. First, our large sample size over multiple years allowed for identification of a smaller yet clinically important subsets of patients for whom future research and improvement initiatives can be targeted. Second, this measure was developed because beyond being relevant to clinical practice, it is also simple and intuitive—requiring only the need to review the patient’s last 24 hour opioid requirements and simple arithmetic. Third, it is a scalable measure that can be implemented by prescribers individually or at a health system level. Given that this measure can be calculated for any inpatient, scalability also includes implementation on other inpatient services beyond internal medicine. Finally, this metric is more patient-specific than the conventional metric and better represents how patients will truly take their opioids once discharged from the hospital.

Our study also has limitations. First, our study does not directly assess association with health outcomes. However, the associations of prescribing prolonged opioid courses with poor patient outcomes and extra opioids in the home with increased opioid misuse by family and friends are already well-established. Second, our measure of opioids prior to admission depended on provider medication reconciliation in the EMR. While it may have slightly changed our comparison only of patients who were and were not on opioids prior to admission, compliance on admission medication reconciliation is generally high and reflects real clinical practice for increased generalizability. Last, this was a single center study, which may limit generalizability. However, this metric is a universal calculation not specific to a given patient population, and thus will certainly be relevant in other health systems.

In summary, these results are important for identifying the subset of patients being discharged from the hospital who are truly high risk and missed by the conventional discharge prescribing metric. This novel metric has potential for impact both on individual patient outcomes and the opioid epidemic more broadly through reduction of excess opioids in the home. We believe that adjusted days metric is more patient-centered and relevant to clinical practice by all accounts, reflecting the reality of how long an opioid prescription dispensed to a patient on discharge will truly last, instead of the providers intent for prescription duration. We also identified two subpopulations of patients receiving high-risk opioid prescriptions: patients receiving liquid opioids, and patients not on opioids prior to admission and not receiving opioids in the 24 hours prior to discharge, but still receiving new discharge opioid prescriptions. Individual providers could avoid these high-risk prescriptions by using the adjusted days metric. Furthermore, we predict a future state where integration of an electronic opioid calculator and clinical decision support tool for this new metric may minimize provider cognitive load, maximize efficiency, and ensure discharge prescription accuracy where true prescription duration matches intended duration, all while offering systematization and standardization of practice.

## Supporting information

S1 Fig Variability of within-subject difference between measurements with magnitude of the mean of within-subject measurements. After log transformation, the within-subject difference between conventional and adjusted days measurements does not vary with the magnitude of within-subject mean of the two measurements, allowing for calculation of mean and standard deviation of within-subject difference that does not vary with magnitude. The solid red line demonstrates the mean log difference between measurements, and the dotted lines represent two log standard deviations from the mean—between which 95% of all measurements fall.
(PDF)

S1 Table Predictors associated with high delta days. Multiple linear regression assessing patient and admission predictors associated with a large difference between adjusted and conventional days measures. * = reference category.
(PDF)

S2 Table Patient predictors associated with receiving an opioid on discharge if not on opioids prior to admission and receiving opioids during hospitalization, but not in 24 hours prior to discharge. * = reference category.
(PDF)
