# Supplementary figures and images for: Evaluation of a novel metric for personalized opioid prescribing after hospitalization
Source: PLoS One. 2020 Dec 31;15(12):e0244735. doi: 10.1371/journal.pone.0244735 (PMC7774844; doi:10.1371/journal.pone.0244735)

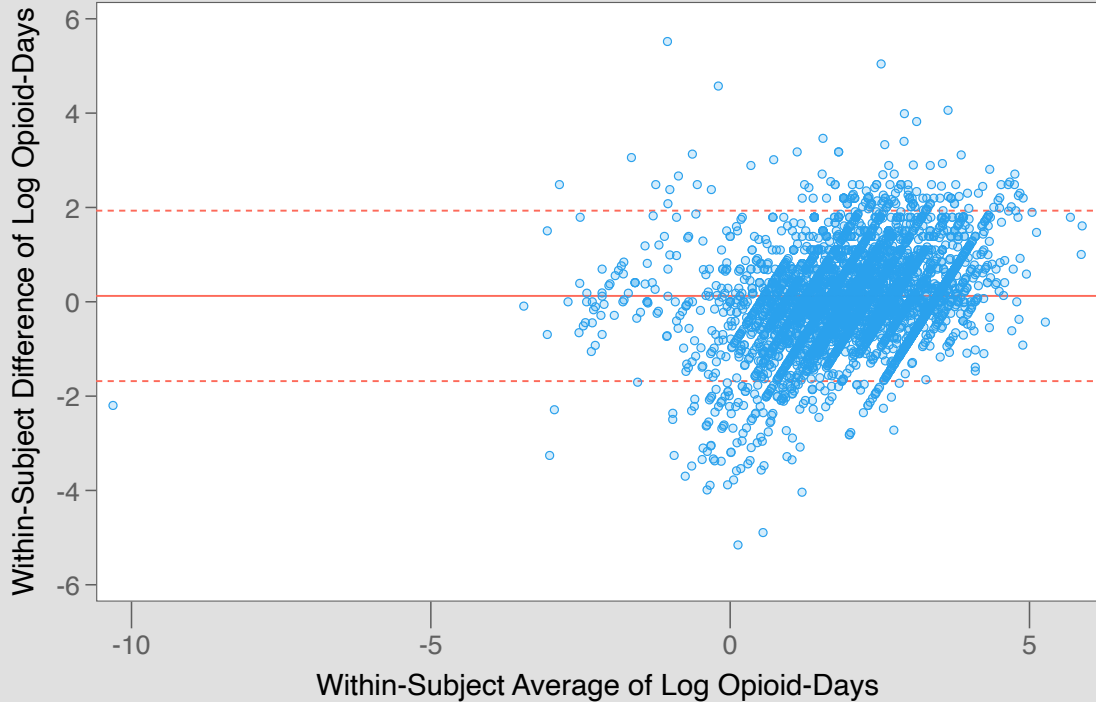

Supplement: S1 Fig — After log transformation, the within-subject difference between conventional and adjusted days measurements does not vary with the magnitude of within-subject mean of the two measurements, allowing for calculation of mean and standard deviation of within-subject difference that does not vary with magnitude. The solid red line demonstrates the mean log difference between measurements, and the dotted lines represent two log standard deviations from the mean—between which 95% of all measurements fall. (PDF) [file pone.0244735.s001.pdf]
