# Supplementary material for: Evaluation of a novel metric for personalized opioid prescribing after hospitalization
Source: PLoS One. 2020 Dec 31;15(12):e0244735. doi: 10.1371/journal.pone.0244735 (PMC7774844; doi:10.1371/journal.pone.0244735)
Supplement: S1 Table — Multiple linear regression assessing patient and admission predictors associated with a large difference between adjusted and conventional days measures. * = reference category. (PDF) [file pone.0244735.s002.pdf]

Covariate	Beta Coefficient	Standard Error	P-value	Delta Days Percent Change	Beta Coefficient 95% Confidence Interval	Percent Change 95% Confidence Interval
Age	0.019	0.003	<0.001	+2.0%	[0.014 – 0.025]	[1.4 – 2.6]
Female	-0.020	0.123	0.874	+1.9%	[-0.262 – 0.223]	[-23.0 – 24.9]
Limited English proficiency	0.185	0.254	0.466	+20.4%	[-0.314 – 0.684]	[-26.9 – 98.3%]
Race/Ethnicity	—	—	—	—	—	—
• *White**	—	—	—	—	—	—
• *Black/African American*	0.125	0.153	0.415	+13.3%	[-0.176 – 0.425]	[-16.1 – 52.9]
• *Latinx/Hispanic*	0.320	0.200	0.111	+37.7%	[-0.073 – 0.714]	[-7.1 – 104.2]
• *Asian*	0.357	0.189	0.059	+42.8%	[-0.014 – 0.727]	[-1.4 – 107.0]
• *Native American or Alaska Native*	-0.901	0.759	0.236	-59.4%	[-2.391 – 0.589]	[-90.8 – 80.2]
• *Native Hawaiian or Pacific Islander*	-0.588	0.585	0.315	-44.5%	[-1.737 – 0.560]	[-82.4 – 75.0]
Mood disorder	-0.232	0.157	0.141	-20.7%	[-0.540 – 0.077]	[-41.8 – 8.0]
Anxiety disorder	0.361	0.189	0.057	+43.5%	[-0.011 – 0.733]	[-1.1 – 108.1]
PTSD	-0.020	0.412	0.962	-2.0%	[-0.828 – 0.789]	[-56.3 – 120.0]
Non-mood psychotic disorder	-0.647	0.390	0.098	-47.6%	[-1.413 – 0.120]	[-75.7 – 12.7]
Benzodiazepine use prior to admission	-0.033	0.141	0.813	-3.3%	[-0.310 – 0.243]	[-26.7 – 27.5]
Liquid opioid use	0.857	0.268	0.001	+135.6%	[0.330 – 1.384]	[39.1 – 299.0]
ICU stay	0.210	0.200	0.293	+23.4%	[-0.182 – 0.603]	[-16.7 – 82.7]
Discharge from teaching service	0.078	0.141	0.582	+8.1%	[-0.199 – 0.355]	[-18.1 – 42.6]
Discharge location	—	—	—	—	—	—
• *Home or self care**	—	—	—	—	—	—
• *Home health care*	0.134	0.140	0.338	+14.4%	[-0.141 – 0.410]	[-13.1 – 50.7]
• *Monitored non-hospital facility*	0.062	0.186	0.739	+6.4%	[-0.303 – 0.427]	[-26.2 – 53.3]
• *Other acute care hospital*	0.029	0.892	0.974	+2.9%	[-1.723 – 1.781]	[-82.2 – 493.4]
• *Against medical advice*	-3.020	1.080	0.005	-95.1%	[-5.132 – -0.907]	[-99.4 – -59.6]
AHRQ mortality index	-0.013	0.008	0.112	-1.3%	[-0.029 – 0.003]	[-2.8 – 0.3]
AHRQ re-admission index	0.012	0.005	0.022	+1.2%	[0.002 – 0.023]	[0.2 – 2.3]
Days to appointment	0.046	0.016	0.004	+4.7%	[0.015 – 0.077]	[1.5 – 8.1]
Date of discharge (by month)	-0.001	0.003	0.630	-0.1%	[-0.007 – 0.004]	[-0.7 – 0.4]
Average daily MME during hospitalization	-0.001	0.000	0.011	-0.0%	[-0.001 – -0.000]	[-0.1 – -0.0]

P-value associated with F-value: <0.001
R-squared: 0.616
Adjusted R-squared: 0.600
Root mean square error: 1.481
