# Supplementary material for: Evaluation of a novel metric for personalized opioid prescribing after hospitalization
Source: PLoS One. 2020 Dec 31;15(12):e0244735. doi: 10.1371/journal.pone.0244735 (PMC7774844; doi:10.1371/journal.pone.0244735)
Supplement: S2 Table — * = reference category. (PDF) [file pone.0244735.s003.pdf]

Covariate	Beta Coefficient	Standard Error	Odds Ratio	P-value	Odds Ratio 95% Confidence Interval
Age	-0.013	0.003	0.99	<0.001	[0.98 – 0.99]
Female	0.051	0.123	1.05	0.676	[0.83 – 1.34]
Limited English proficiency	0.208	0.177	1.23	0.239	[0.87 – 1.74]
Race/Ethnicity	—	—	—	—	—
• *White**	—	—	—	—	—
• *Black/African American*	-0.411	0.211	0.66	0.051	[0.44 – 1.00]
• *Latinx/Hispanic*	-0.361	0.211	0.70	0.087	[0.46 – 1.05]
• *Asian*	-0.158	0.168	0.85	0.345	[0.61 – 1.19]
• *Native American or Alaska Native*	0.595	0.692	1.81	0.390	[0.47 – 7.04]
• *Native Hawaiian or Pacific Islander*	-0.163	0.458	0.85	0.722	[0.35 – 2.09]
Mood disorder	-0.661	0.244	0.52	0.007	[0.32 – 0.83]
Anxiety disorder	-0.003	0.268	1.00	0.993	[0.59 – 1.69]
PTSD	-0.962	1.127	0.38	0.393	[0.04 – 3.48]
Non-mood psychotic disorder	-1.256	0.537	0.28	0.019	[0.10 – 0.82]
Benzodiazepine use prior to admission	0.278	0.199	1.32	0.162	[0.89 – 1.95]
ICU stay	-0.851	0.175	0.43	<0.001	[0.30 – 0.60]
Discharge from teaching service	-0.994	0.132	0.37	<0.001	[0.29 – 0.48]
Discharge location	—	—	—	—	—
• *Home or self care**	—	—	—	—	—
• *Home health care*	0.487	0.172	1.63	0.005	[1.16 – 2.28]
• *Monitored non-hospital facility*	1.146	0.170	3.15	<0.001	[2.25 – 4.39]
• *Other acute care hospital*	0.756	0.425	2.13	0.076	[0.92 – 4.90]
• *Against medical advice*	-0.956	1.024	0.39	0.350	[0.05 – 2.86]
AHRQ mortality index	-0.002	0.010	1.00	0.856	[0.98 – 1.02]
AHRQ re-admission index	-0.007	0.006	0.99	0.284	[0.98 – 1.01]
Date of discharge (by month)	-0.019	0.003	0.98	<0.001	[0.98 – 0.99]
Average daily MME during hospitalization	0.000	0.001	1.00	0.727	[1.00 – 1.00]

Likelihood ratio chi-square test: 139.5
P-value for chi-square: <0.0001
Pseudo R-squared: 0.0669
